# Assessment of Genetic Relationships between *Streptocarpus x hybridus* V. Parents and F1 Progenies Using SRAP Markers and FT-IR Spectroscopy

**DOI:** 10.3390/plants9020160

**Published:** 2020-01-28

**Authors:** Monica Hârţa, Orsolya Borsai, Cristina M. Muntean, Nicoleta E. Dina, Alexandra Fǎlǎmaş, Loredana Elena Olar, Katalin Szabo, Doru Pamfil, Răzvan Ştefan

**Affiliations:** 1 Life Sciences Institute, Research Centre for Biotechnology in Agriculture affiliated to Romanian Academy, University of Agricultural Sciences and Veterinary Medicine Cluj-Napoca, 3-5 Mănăştur St., 400372 Cluj-Napoca, Romania; monica.harta@usamvcluj.ro (M.H.); katalin.szabo@usamvcluj.ro (K.S.); dpamfil@usamvcluj.ro (D.P.); 2 AgroTransilvania Cluster, Dezmir, Crișeni St., FN, Dezmir 407039, Romania; 3 National Institute for Research and Development of Isotopic and Molecular Technologies, 67-103 Donat St., 400293 Cluj-Napoca, Romania; nicoleta.dina@itim-cj.ro (N.E.D.); alexandra.falamas@itim-cj.ro (A.F.); 4 Life Sciences Institute, Research Centre for Biophysics, University of Agricultural Sciences and Veterinary Medicine Cluj-Napoca, Faculty of Veterinary Medicine, 3-5 Mănăştur St., 400372 Cluj-Napoca, Romania; olarloredanaelena@yahoo.com (L.E.O.); rstefan@usamvcluj.ro (R.Ş.)

**Keywords:** cape primrose, F1 hybrids, gDNA, multivariate analysis, discrimination

## Abstract

The genetic relationship among three Streptocarpus parents and twelve F1 hybrids was assessed using sequence-related amplified polymorphism (SRAP) molecular markers and Fourier-transform infrared (FT-IR) spectroscopy. Both methods were able to discriminate F1 hybrids and parents as revealed by cluster analysis. For hybrid identification, the type III SRAP marker was the most effective due to the presence of male-specific bands in the hybrids. Different behaviors in the biochemical variability of DNA samples have been observed by FT-IR spectral analysis, which might be attributed to the inherent nature of the genomic DNA from parents and their F1 progenies. Mantel test was also carried out to compare morphological, SRAP, and FT-IR results based on genetic distances. The highest correlation coefficient was found between morphological and SRAP marker distances (R = 0.607; *p* ≤ 0.022). A lower correlation was observed between the morphological and FT-IR distance matrix (R = 0.231; *p* ≤0.008). Moreover, a positive correlation was found between the distances generated with SRAP and FT-IR analyses (R = 0.026) but was not statistically significant. These findings show that both SRAP and FT-IR techniques combined with morphological descriptions can be used effectively for nonconventional breeding programs for Streptocarpus to obtain new and valuable varieties.

## 1. Introduction

Streptocarpus, known as Cape primrose, belongs to the Streptocarpus subgenus, being extensively cultivated worldwide as ornamental plants due to their beautiful flowers [1,2]. Similar to other horticultural species, the hybridization followed by phenotypic selection was the most commonly used breeding method also for Cape primrose varieties. The characterization of hybrids was generally based on morphological traits [3]. Combining conventional selection with marker-assisted selection (MAS) can enable the identification and characterization of the hybrids more precisely and effectively [4].

The first genetic map in Streptocarpus genus has recently been generated with RAD (restriction- site associated DNA) sequencing-based SNP (single nucleotide polymorphism) markers [5], providing valuable information related to linkage groups between DNA markers and traits. However, the establishment of the genetic relationships between morphological traits and DNA molecular markers could represent, in some cases, an alternative to genetic mapping and could be economically useful for the characterization and identification of F1 hybrids [6]

In this context, DNA-based molecular marker methods represent valuable tools for genetic relationship evaluation among cultivars and species to many ornamental plants [7,8,9,10,11,12,13,14,15,16,17]. Among these methods, SRAP (sequence-related amplified polymorphism) is recognized to be a useful and inexpensive molecular marker technique for hybrid identification and genetic relationship studies [18,19,20] as compared to other approaches based on next generation sequencing (NGS) techniques [5]. Furthermore, Fourier-transform infrared (FT-IR) spectroscopy is also an easy, low-cost, and rapid method for analyzing the molecular content of complex samples based on the vibrations of both functional groups and highly polar bonds of sample components even on a large scale [21,22]. It can easily be applied also for hundreds of progenies, since the only chemical substance used is KBr, which can be purchased at accessible price. The quantity of KBr used for one sample is low (0.200 g), which makes it possible to prepare hundreds of samples. Regarding the efficiency of this method, around 40–50 samples can be analyzed/day.

Based on FT-IR spectra, the chemical composition of genomic DNA can be identified and some of the spectral bands can be assigned to distinct chemical substructures or specific functional groups [23].

The differences among spectral bands might be directly related to subtle changes in the base structural groups and backbone structures of genomic DNA from different plant samples, such as hybrids and their parents [24]. Thus, considering these results, it can be stated that FT-IR spectral analysis could be applied as a useful tool to distinguish F1 hybrids from their parents based on structural variability of plant genomic DNA. In general, DNA structural changes might be responsible for modifying the biological function of these nucleic acids.

Several research articles have already been published on the characterization of F1 Streptocarpus progenies at the molecular level. Hughes et al. (2004) [25] studied the mode of chloroplast DNA inheritance on F1 hybrids from reciprocal crosses between *S. primulifolius* and *S. aff. Primulifolius*, and between *S. rexii* and *S. dunnii*, based on trnL/trnF intergenic spacer length polymorphism. However, to the best of our knowledge, publications are rare or missing on the evaluation of the genetic relationships among Cape primrose hybrids using nuclear DNA-based markers and no comparisons were made between SRAP markers and FT-IR spectral data regarding the efficiency and usefulness of the two different techniques. Therefore, the main aim of this research was to identify genetic variations between genitors and their cross-pollinated Streptocarpus F1 progenies at the molecular level by using both the SRAP and FT-IR techniques. The application of these techniques in nonconventional breeding programs could facilitate the selection of the initial breeding material to obtain new and valuable Streptocarpus varieties. To select the genotypes with the highest ornamental potential, a detailed description of the F1 progenies was carried out based on the morphological traits of the flowers.

## 2. Results

### 2.1. Pollen Viability

Between the two male parents, no considerable differences in pollen viability were observed. The percentages for “Slumber Song” and for “Snow White” were 64.5% and 60.5%, respectively, which allowed to proceed to hybridization. Similar viability percentages were recorded by Afkhami-Sarvestani et al. (2012) [26], who tested pollen viability for interspecific crosses within the Streptocarpus subgenus Streptocarpella.

### 2.2. Morphological Characterization and Genetic Relationships Between Parents and F1 Progenies

**Figure 1:**
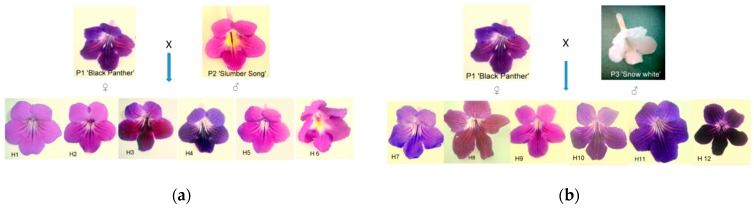
Flower colors of the Streptocarpus progenies due to cross pollination: (**a**) flower color of the F1 progenies (H1–H6) from the parental cross P1 × P2 (♀ “Black Panther” × ♂ “Slumber Song”) and (**b**) flower color of F1 progenies (H7–H12) from the parental cross P1 × P3 (♀ “Black Panther” × ♂ “Snow White”).

**Figure 2:**
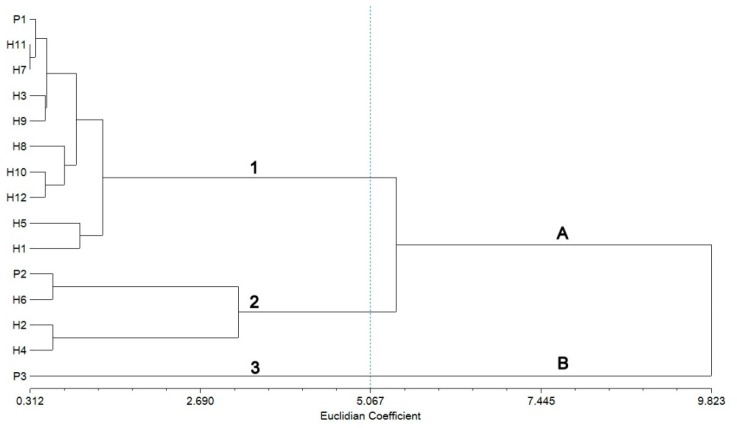
UPGMA morphological dendrogram showing the relationships between Streptocarpus parents and F1 hybrids.

As shown in Table S1, different flower characters were examined for the genitors and F1 progenies. The results show that the highest average numbers of flowers (20.87 ± 0.33 and 21.07 ± 0.31) have been recorded respectively in H3 and H10 obtained from P1 × P2 and P1 × P3 parental crosses. The smallest average numbers of flowers (16.40 ± 0.23) were observed in H6 (16.33 ± 0.47) and H9 (20.47 ± 0.23). Furthermore, the highest and lowest average numbers of penducle/plant (9.40 ± 0.23 vs. 7.40 ± 0.23) were recorded respectively in H1 and H6, both resulting from the P1 × P2 parental cross. Another important parameter analyzed was the number of flowers/peduncles, a very good indicator of ornamental value. Therefore, from the first parental cross (P1 × P2), H3 showed the highest average number of flowers/peduncles (2.43 ± 0.07) while H5 revealed the lowest average number of flowers (2.16 ± 0.09) among the progenies. The highest average flower width (6.64 ± 0.05) was recorded in H12, resulting from the P1 × P3 parental cross, while the lowest average flower width was observed in H6 (4.16 ± 0.04), obtained from P1 × P2. After flower size, flower color is one of the most important characters which can improve the ornamental value of any plant. The results show that differences in terms of petal color of the hybrid plants are minor since all the hybrids had in common combinations of pink and violet color in their petal coloration (Figure 1 and Table 1). Regarding genetic relationships between the parents and F1 hybrids, the results of the built UPGMA (unweighted pair-group method with arithmetic mean) dendrogram showed a high goodness of fit according to Mantel’s test (r = 0.932; approximate Mantel t-test: t = 4.716; probability random Z < observed Z: *p* = 1.000) and two main groups (A and B) were evidenced based on the Euclidian distance (Figure 2). Nine of the F1 hybrids clustered in group A with P1 except H2, H4, and H6, which were grouped with P2.

### 2.3. Assessment of Genetic Relationships based on SRAP Analysis

Our results show that SRAP markers were suitable to assess the genetic relationships between parents and F1 hybrids. Out of the 32 primer combinations screened in this study for their ability to amplify the DNA samples from *S. x hybridus* V., eight revealed reproducible and consistent results. The other 24 primer combinations either generated monomorphic bands or did not produce PCR amplification for all the analyzed samples. The levels of polymorphism detected with the eight selected SRAP primer pairs are presented in Table 2.

The eight SRAP primer combinations amplified 96 reproducible fragments ranging in size from 104 to 1949 bp, out of which 90 bands were polymorphic bands. The number of polymorphic bands for each primer pair ranged from 7 to 15. The highest number of polymorphic bands (15) was generated by the Me1-Em2 primer combination, whereas the lowest number of amplified polymorphic bands (7) was obtained with the Me1-Em8 primer combination. The percentage of polymorphism ranged from 88.88% (Me8-Em2) to 100% (Me1-Em8 and Me6-Em8) with a mean percentage of 93.75%. As it is known, PIC (polymorphic information content) is a measure of allele frequencies at single loci or summed multiple loci [27]. In the present study, the PIC values for each primer combination ranged from 0.34 (Me8-em2) to 0.48 (Me1-Em2), with an average number of 0.4, indicating that the selected SRAP markers were able to identify a high degree of polymorphism (Table 2). For dominant markers, such as SRAP markers, the PIC values ranged from 0 to 0.5, where 0 indicates the fixation of one allele and 0.5 means equal frequencies of alleles [28]. Based on the band profile analysis in hybrids and parents, SRAP markers were classified into three different types. A discriminative analysis of the inheritance of the single locus for the hybrids and parents from each cross was performed based on the polymorphism revealed by SRAP marker combinations. The bands that were common in F1 hybrids and both parents were considered type I, and bands that were common in F1 hybrids and one parent were considered type II (female parent) and type III (male parent), respectively, as shown in Table 3.

Among the three different types of markers obtained, type I and type III were more frequent in P1 × P2 compared to P1 × P3 from the total occurrence of each type of SRAP markers. In case of the P1 × P3 parental combination, type I and type II markers were more frequent. The difference between the total numbers of occurrences for each type of SRAP markers in each parental cross was observed and generated valuable information about the number of SRAP bands inherited from male parents compared to those inherited from the female parent.

### 2.4. Cluster Analysis Revealed by SRAP Markers

**Figure 3:**
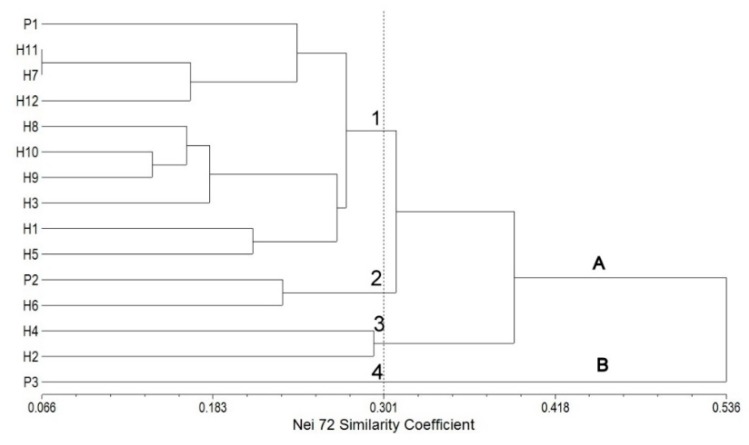
UPGMA dendrogram of Streptocarpus parents and F1 based on Nei72′s similarity coefficient.

In the SRAP analysis, the UPGMA dendrogram grouped all the Streptocarpus parents and hybrids into two main clusters (marked A and B). Similar to the results obtained by cluster analysis based on morphological traits, the first group included both parents (P1 and P2) and hybrids while the second one included only the third parent (P3) used in the second cross. A high cophenetic correlation coefficient of 0.91 was obtained, showing a good fit [29]. The analyzed parents and hybrids clustered into four subgroups at Nei72′s similarity coefficient [30] (pp. 283–292) of 0.301 (Figure 3). 

The first subgroup (from P1 to H5) consists of the P1 (♀ parent) and F1 progenies from P1 × P2 (H1, H3, and H5) and P1 × P3 (H7, H8, H9, H10, H11, and H12) crosses, respectively. Noticeable is the clustering of the two hybrids H11 and H7 on the same line. Regarding the second subgroup, it is noteworthy that P2 (♂ parent) was clustered together with H6. The reason for this grouping might be the similarity of some morphological traits as it can be seen in Table 1. Hybrids H4 and H2 were clustered in the third subgroup. 

### 2.5. FT-IR Spectral Data Combined with Multivariate Analysis

**Figure 4:**
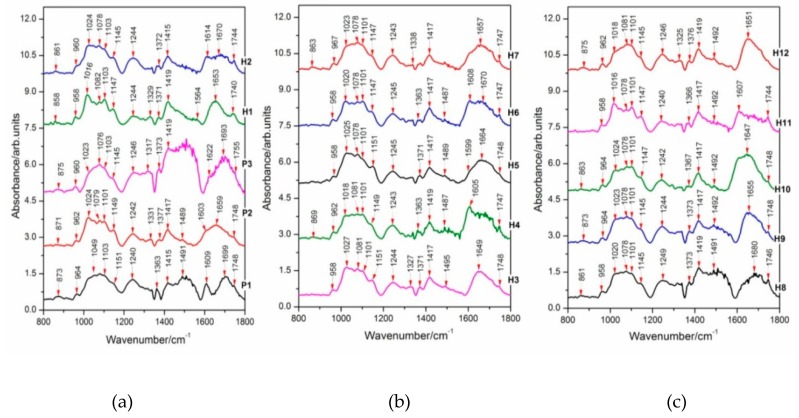
FT-IR absorbance spectra of DNA extracted from different Streptocarpus parents and their hybrids: (**a**) P1, P2, P3, H1, and H2; (**b**) H3, H4, H5, H6, and H7; and (**c**) H8, H9, H10, H11, and H12. All spectra were acquired over 256 scans. Spectra were scaled to have the same intensity in the 1101 cm^−1^ (υ_s_PO_2_^−^) band. Profiles are presented in the wavenumber range 800–1800 cm^−1^**.**

**Figure 5:**
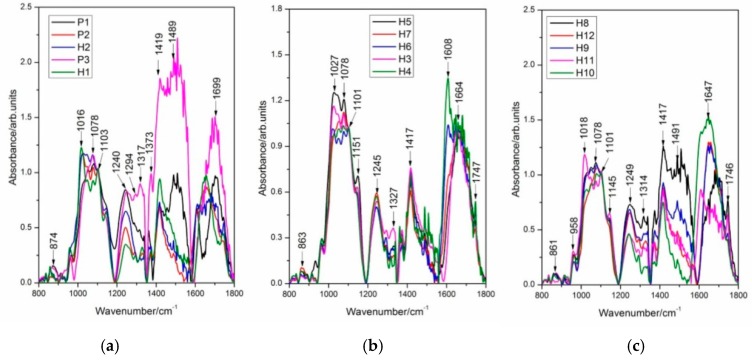
Intensity changes in FT-IR absorbance spectra of DNA extracted from different Streptocarpus parents and hybrids, as labeled in the figure: Spectra were scaled to have the same intensity in the 1101 cm^−1^ (υ_s_PO_2_^−^) band. Profiles are presented in the wavenumber range 800–1800 cm^−1^.

In this study, discrimination between Streptocarpus parents and their F1 hybrids was also assessed by Fourier-transform infrared (FT-IR) spectroscopy in combination with multivariate analysis. Here, FT-IR spectral fingerprints of *Streptocarpus* DNAs are present in the region 800–1800 cm^−1^, as shown in Figure 4 and Figure 5. Labels indicate wavenumber values for the prominent bands in each spectrum (cm^−1^ units). Different color hues represent different values of the displayed spectra. The color of the spectral lines have the only role in facilitating better differentiation between the spectra with no relations between Figure 4a–c.

The spectra in Figure 4 and Figure 5 indicate broad spectral features, covered by overlapping narrow bands. Band intensity changes and wavenumber shifts are to be found in the spectra presented in Figure 4 and Figure 5, being a proof for structural variations in genomic DNA.

Additional data are given in Table 4 and Table 5, based on the peak positions of vibrational markers (FT-IR) showing a comparative analysis of the FT-IR absorbance modes of genomic DNA between the two parental crosses (P1 × P2 and P1 × P3) and their F1 hybrids. Proposed FT-IR band assignments found in the scientific literature for similar compounds are also included. Infrared wavenumber shifts with values above the spectral resolution (4 cm^−1^) might be a proof for structural differences between parents and hybrids and among hybrids. In this study, we analyze also the main bands in the FT-IR absorbance spectra, characterizing the Streptocarpus DNA from parents and F1 hybrids (see the Discussion section). 

**Figure 6:**
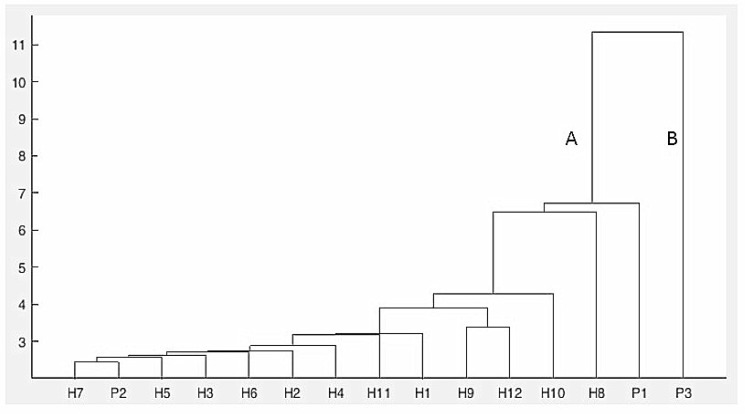
Dendrogram of the three Streptocarpus parents and their F1 hybrids derived from hierarchical cluster analysis (HCA) cluster analysis of the FT-IR spectra.

For multivariate analysis, the 800 to 1800 cm^−1^ region of the FT-IR spectral data was subjected to hierarchical cluster analysis (HCA) and principal component analysis (PCA). The HCA dendrogram following the analysis performed on the FT-IR spectra illustrates the arrangement of the parents and their hybrids and is presented in Figure 6. A high cophenetic correlation coefficient of 0.93 was obtained in this case. This result reveals that there is a good fit between the original similarity matrix and the built dendrogram.

**Figure 7:**
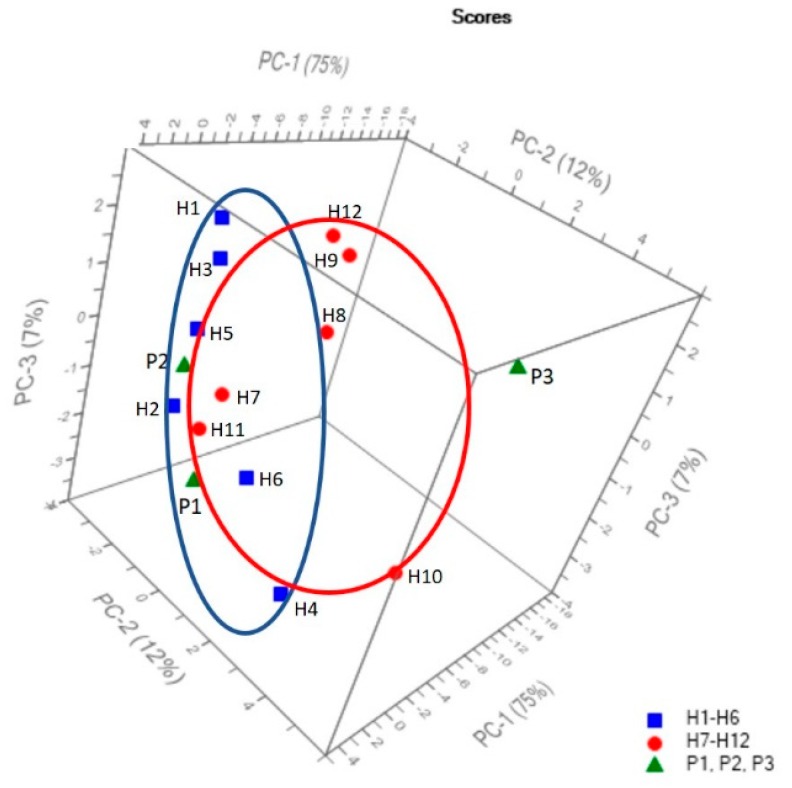
Principal component analysis (PCA) score 3D plots of PC-1 vs. PC-2 and PC-3 obtained for the 15 FT-IR spectra corresponding to the P1–P3 and H1–H12 samples, respectively: P1–P3 and H1–H12 are labelled for clear visualization.

The results show that, in this study, only one out of 12 total spectra for F1 progeny was not correctly predicted. In order to examine the relationships and differentiations between Streptocarpus parents and their F1 hybrids, the preprocessed spectral data were introduced into an ASCII database and used further for PCA analysis. In this study, seven principle components (PCs) were calculated using the Unscrambler X^®^ 10.4 (CAMO Software, Norway), out of which the first three brought the most significant contribution to the total variance (94%), and were chosen to be plotted as 3D PCA scores. Figure 7 depicts the PCA score 3D plots of PC-1 vs. PC-2 and PC-3 obtained for the 15 FT-IR spectra corresponding to the samples. PC-1 explains 75% of the total variance, while PC-2 explains another 12% and PC-3 explains the rest of the 7% total variance.

### 2.6. Comparison of Molecular Marker Systems

A Mantel test analysis was carried out to compare morphological, SRAP, and FT-IR molecular marker systems based on genetic distances. The highest correlation coefficient was found between the distances based on morphologically analysed traits and SRAP markers (R = 0.607; *p* ≤ 0.022). There was a lower correlation between the distance matrix obtained with morphological markers and FT-IR markers (R = 0.231; *p* ≤ 0.008). Moreover, a positive correlation was found between the distances generated with SRAP and FT-IR analysis (R = 0.026) but was not statistically significant.

## 3. Discussion

As shown in supplementary file (Table S1), morphological characterization of the flowers of genitors and F1 progenies was carried out to evaluate the ornamental value of the new progenies. The heredity of color in several species has been studied previously in other ornamental plants such as chrysanthemum [45], roses [46], and alstroemeria [47]. The results of these reports suggest that flower color polymorphism is due to variation in floral pigments, especially anthocyanin (slight changes and single mutations in the anthocyanin pathway) and a predominance of pink, purple, and blue with white combinations [48] (pp. 381–458). It is worth mentioning that none of the hybrids from the P1 × P3 parental cross showed white petals. An explanation for this phenomenon could be that “Snow White” (P3) used for the second parental cross is a diploid X-ray irradiated mutant from “Maassen’s White”—a mutation of the “Constant Nymph”, which has violet-blue flowers. Regarding the other flower traits analyzed in this study, it can be observed that the F1 hybrids exhibited intermediate or superior values compared to their genitors within the analyzed morphological traits but that statistically significant differences were recorded only for certain characters and only for some hybrids. For example, the flower color was variable showing different combinations of pink and violet tones compared to genitors. Interestingly, only one of the hybrids (H6) from the P1 × P2 parental cross showed strong yellow throat such as in P2. Streptocarpus plants are highly appreciated for their flower color, size of the flowers, and peduncle length, but the number of flowers per peduncle plays an important role in the estimation of ornamental value of any individual Streptocarpus plant. In this context, the results show that H5 and H1 had lower average numbers of flowers/peduncles than P2. H2, H3, H4, and H6 had intermediate average flower numbers/peduncles compared to their parents, their number ranging from 2.32 ± 0.07 to 2.35 ± 0.05. From the second parental cross (P1 × P3), all the hybrids presented intermediate values of average flower numbers/peduncles as compared to their parents. 

To evaluate the genetic relationships between parents and F1 progenies, cluster analysis of biometrical measurements was used. The results of the cluster analysis show that hybrids H2, H4, and H6 were clustered in the same subgroup, having similar color (violet) but different color codes (pink violet—N77B and violet—N82D) according to the Royal Horticultural Society Color Chart (RHSCC) scale. The second group (B) included only the third parent (P3) used in the second cross. It is worth mentioning that most of the F1 hybrids morphological characters were very similar to their mother plants. Oehlkers (1964) [49] (pp. 329–370) in his report claims that, in Streptocarpus, many characters have been proven to be controlled by chromosomal genes and specific cytoplasmic components. 

Regarding SRAP analysis, examination of the banding patterns of the two crosses suggests different parental contributions. The type I marker was mostly identified in the P1 × P2 cross, while the type II marker was more frequent in the P1 × P3 cross (Table 3). For hybrid identification, the type III marker especially was very effective and unambiguously identified the true hybrid because of the presence of male-specific bands in hybrids [50]. Information is scant regarding the Streptocarpus genome; thus, it is very hard to predict the number of genes involved in the inheritance of the morphological traits that we have been analyzed. Hence, in the future, it could be of high interest to employ different types of DNA molecular markers to assess more precisely the valuable morphological traits of the Streptocarpus varieties. Our results are in accordance with other reports suggesting that SRAP primer pairs may amplify some nonnuclear DNA fragments and that a greater maternal contribution has also been observed in hybrids (Paeonia sp., Zyosia sp., and Coffea arabica) when SRAP markers were used, as reported by Hao et al. (2008) [18], Xuan et al. (2008) [50], and Mishra et al. (2011) [51].

In this work, discrimination between Streptocarpus parents and their F1 hybrids was also evidenced by Fourier-transform infrared (FT-IR) spectroscopy. To reach this goal, we have analyzed the main bands in the FT-IR absorbance spectra, characterizing the Streptocarpus DNA from parents and F1 hybrids. The region between 700–900 cm^−1^ was found to involve characteristic modes arising from sugar vibrations or from a coupling of the sugar pucker mode with the phosphate group motions [52]. We have found a band centered near 874 cm^−1^ in the FT-IR absorbance spectra of genomic DNA presented in Figure 5a, showing a contribution of the phosphodiester backbone and deoxyribose with a sugar geometry of N type (possible A-form) [31,32,33,34]. The region between 1000–1250 cm^−1^ contains information about the phosphate and sugar absorptions. According to Lindqvist and Gräslund (2001) [53], these vibrations could generate high-intensity marker profiles for backbone conformations. An infrared absorbance band centered near 1016 cm^−1^ from DNA samples is shown in Figure 5a, indicating contributions arising from deoxyribose [32]. Moreover, significant changes were observed for the 1078 cm^−1^ PO_2_^−^ symmetric stretching vibration (υ_s_PO_2_^−^) of DNA. Similar results were also reported by Pevsner and Diem (2001) [37], Andrushchenko et al. (2003) [38], and Muntean et al. (2013) [31]. Subtle intensity changes in FT-IR absorbance spectra of the DNA samples were found in the region of 800–1200 cm^−1^ as compared to the rest of FT-IR profiles (1200–1800 cm^−1^) and are presented in the Figure 5a–c. A mode which shows large variations in intensity was located near 1240 cm^−1^ and is assigned to PO_2_^−^ antisymmetric stretch [31,32,40]. This band represents a possible marker band of A-form DNA in the spectra of the analyzed samples (Figure 5a). In the wavenumber range 1250–1500 cm^−1^, bands due to the base-sugar entities strongly dependent on the glycosidic torsion angle can be observed [40]. In this interval, the band appearing near 1373 cm^−1^ in our study denotes contributions from the dA and dG residues (C2′-endo/anti). Similar results were also reported by several authors [32,39,41]. Another strong peak appeared near 1419 cm^−1^ and is attributed to the C2′-endo/anti sugar pucker (B-form) [32,39]. Furthermore, in the region of 1500 cm^−1^–1800 cm^−1^, vibrations sensitive to base pairing and base stacking interactions appeared as previously reported by Lindqvist and Gräslund (2001) [53].

The spectral signature of P3 is highly different as compared to the other FT-IR spectra of DNA samples from parents and hybrids. The spectral response of DNA from the hybrids H3–H7 is almost identical in the wavenumber range 1200–1500 cm^−1^, belonging to the base-sugar entities. Significant structural differences are put into evidence between P1, P2, P3, H1, and H2 (Figure 5a) and between H8, H9, H10, H11, and H12 (Figure 5c), as judging from the analysis of the spectral interval 1200–1800 cm^−1^, characterizing base-sugar structures, base ring modes, and base pairing and base stacking interactions.

As shown in Figure 6, the HCA dendrogram organized all of the samples in two main clusters (A and B). The first cluster (from P1 to H7) consists of P1 (♀ parent), P2 (♂ parent), and the F1 progenies, both from the P1 × P2 and P1 × P3 crosses. The second major branch of the dendrogram consisted of P3 parental genitor. Our results show that the HCA dendrogram could discriminate the F1 progeny and their genitors, although a very few hybrids were not correctly clustered (e.g., P2 was not used as genitor for the H7).

**Figure 8:**
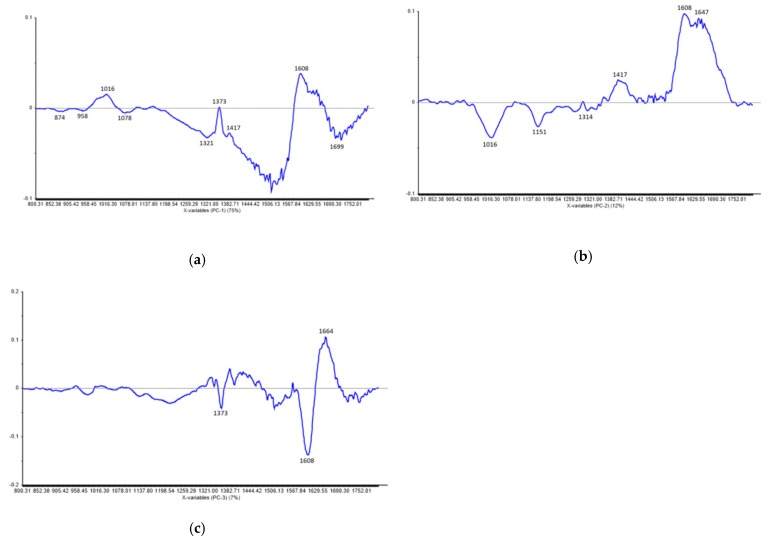
Principal components loadings showing the marker bands considered as the main contribution in the grouping of the spectral data: first principle component (PC) (**a**), second PC (**b**), and third PC (**c**).

As shown in Figure 7, a grouping tendency can be noticed for H1–H6 and H7–H12 samples, together with the P1 and P2 parent samples. The third parent (P3) is isolated and considered an “outlier” not being grouped with the others. However, the two marked groups overlap as a result of their input data similarities in terms of band relative intensity, corresponding wavenumbers, and spectral profile. Our results are in accordance with the results derived from a previous research carried out by Song et al. (2014) [24], who stated that discrimination of F1 progenies based on FT-IR spectral similarity can be explained by their genomic similarity. This result was expected as the two groups of samples originated from the same parent (P1). Furthermore, Muntean et al. (2009) [54], in one of their report, claimed that similarity between the spectral differences of the genomic DNA confirms a positive taxonomic relationship between higher plants. The loadings for PC-1, PC-2, and PC-3 are plotted in Figure 8 and have a spectral profile, providing most of the observed FT-IR bands (874 cm^−1^, 958 cm^−1^, 1016 cm^−1^, 1078 cm^−1^, 1151 cm^−1^, 1314 cm^−1^, 1321 cm^−1^, 1373 cm^−1^, 1417 cm^−1^, 1608 cm^−1^, 1647 cm^−1^, 1664 cm^−1^, and 1699 cm^−1^) as maxima or minima which contribute to the grouping of the input spectral data.

The characteristic FT-IR spectral differences resulting from this study could be attributed to the inherent nature of genomic DNA from parents and their F1 progenies. In addition, DNA recombination during sexual reproduction might influence the occurrence of distinct spectral variations in the analyzed genomic DNA samples from Streptocarpus parents and their F1 hybrids.

## 4. Materials and Methods

### 4.1. Plant Material

In this study, three *Streptocarpus x hybridus* V. varieties were used as parents for artificial hybridization. “Black Panther”, “Slumber Song”, and “Snow White” were selected as genitors based on commercial and ornamental values, especially, flower characteristics. The genitors were purchased from two certified nurseries from North Wales, UK. “Black Panther” is an American variety created by John Ford (1982) [55] with dark purple flowers and two thin yellow bars from the throat of each flower. “Slumber Song” is another variety created also by J. Ford with medium purple color of the corolla, pink splashes on all petal lobes, and strong yellow throat. “Snow White” is a variety derived from “Maassen’s White” with white corolla and almost erect calyx and is considered a “miniature sport”. “Black Panther” (P1) was used as a common maternal plant, while “Slumber Song” (P2) and “Snow White” (P3) cultivars were used as paternal plants for two crosses (P1 × P2 and P1 × P3). All the crosses were made in July 2013, and the newly produced seeds were collected after three months. The seeds (cca. 80 seeds/cross) obtained from the twisted dried capsules were sown in October 2013 in polystyrene boxes with standard sowing substrates. The boxes were kept at 26 °C with a high relative humidity of 98% in the greenhouse. After two months, the seedlings were transferred to plastic pots with a diameter of 9 cm and grown as potted plants in greenhouse conditions. The first blooming was recorded June 2014.

### 4.2. Pollen Viability Test

Fresh pollen grains from anthers were collected at anthesis, dried, and stored at 4 °C before the germination test was performed. The viability of pollen grains for each father cultivar (“Slumber Song” and “Snow White”) was determined using in vitro pollen germination test according to the protocol of Afkhami-Sarvestani et al. (2012) [26].

### 4.3. Cross Pollination Between Parents and F1 Progeny Selection

Artificial pollination was performed on a single mother plant. The flower buds of the maternal plant (“Black Panther”) were emasculated at the early bud stage. Hand pollination was performed 5 days after emasculation when the stigmas had begun to excrete secretion. Ten flowers of the mother plant were pollinated with ♂ ”Slumber Song”, and the other ten were pollinated with ♂ “Snow White”.

The pollinated flowers for each cross (♀ “Black Panther” × ♂ “Slumber Song” and ♀ “Black Panther” × ♂ “Snow White”) were bagged immediately after pollination. In order to save all the seeds, shortly before maturity, the capsules were isolated using a paper bag and closed by clips. The twisted capsules were harvested after reaching full maturation.

In greenhouse conditions, hybridization of P1 × P2 resulted in 19 F1 flowering progenies and P1 × P3 produced 14 F1 flowering progenies. It is important to mention that some of the F1 flowering progenies of each cross showed no morphological variation in terms of flower characteristics, especially petal colors. For this reason, an initial selection was applied.

The initial selection of the progenies for further analyses was based on visual observations considering their capacity to produce a higher or intermediate number of flowers/plants compared to their parents. Flower color (FC) was considered also an important trait which highly defines the ornamental value of the plants. Petal colors were identified using the Royal Horticultural Society Color Chart (RHSCC) based on visual comparisons. Flower color of the parent plants and F1 progenies selected for molecular analysis are presented in Figure 1 and Table 1. In order to carry out the molecular analysis, six F1 Streptocarpus progenies from each cross (♀ “Black Panther” × ♂ “Slumber Song” and ♀ “Black Panther” × ♂ “Snow White”) were selected based on their ornamental values. Meanwhile, the same individuals were propagated by leaf cuttings and grown in greenhouse conditions to ensure the data set for morphological analyses was organised in a completely randomised block design (CRBD) in three replications.

Five plants per F1 progeny and each parental plant propagated by leaf cuttings were selected for morphological data analysis. The propagated plants presented the same morphological characters as the mother plant.

The morphological traits measured were the following: number of flowers/plant (NF), number of peduncles/plants (NP), number of flowers/peduncles (FP), length of peduncle (LP), length of corolla tube (LCT), and width of flower (WF). The width of flower was measured perpendicular to the plane of symmetry, at the widest part according to Lázaro and Totland [56] for zygomorphic flower characterization. Data were recorded starting from the first bloom for 120 days. Blooming was continuous with its peaks in June and August 2015.

All selected plants for morphological analysis presented a uniform and identical flower coloration pattern to the plants that were initially selected. To carry out SRAP and FT-IR analyses, one individual (propagated by leaf cuttings) from every parent and F1 progeny was randomly selected and subjected to analyses.

### 4.4. DNA Isolation

Young Streptocarpus leaves were harvested from each parent and F1 progenies. The total DNA was isolated using the CTAB-based method as published by Lodhi et al. (1994) [57] and improved by Pop et al. (2003) [58] and Bodea et al. (2016) [59]. DNA purity and concentration were determined with a NanoDrop-1000 spectrophotometer (Thermo Fisher Scientific, Waltham, USA). To perform SRAP analysis, DNA samples were diluted to 50 ng/µl using distilled water. The final concentration of the genomic DNA used for FT-IR spectroscopy was 5 mg/µl. The purity of the genomic DNA (calculated as absorbance ratios OD260/OD280) was also evaluated before the analysis to minimize the detrimental effect of DNA contamination.

### 4.5. SRAP Analyses

In the present study, thirty-two different primer combinations were tested for PCR amplification including 4 forward and 8 reverse primers (Table 6).

The primer combinations were screened on all the analysed samples. Subsequently, 8 primer combinations that generated clearly reproducible DNA bands and presented high levels of polymorphism were chosen for further analyses. Reaction mixtures (total volume of 15 μL) contained 50 ng genomic DNA, 0.3 µM of each primer (Kaneka-Eurogentec, Belgium), 1.5 mM of MgCl_2_, 0.2 mM of dNTPs, 5x green PCR buffer, 1 U Go Taq DNA polymerase (Promega, Madison, Wisconsin, USA), and nuclease-free water (Sigma-Aldrich GmbH, Germany). DNA amplification was carried out in a 96-well Gradient Palm-Cycler (Corbett Research, UK) using the PCR program as described by Li and Quiros (2001): 5 min of denaturation at 94 °C, five cycles of 1 min of denaturation at 94 °C, 1 min of annealing at 35 °C, and 1 min of elongation at 72 °C and then 35 cycles (94 °C for 1 min; 50 °C for 1 min, and 72 °C for 1 min) with a final elongation step of 10 min at 72 °C. Separation of the amplified products was performed on 2% agarose gels (Promega, Madison, Wisconsin, USA) in 1xTAE, at 0.29 V/cm^2^ for 2 h. The molecular marker used was 100 bp DNA Step Ladder (Promega, Madison, Wisconsin, DC, USA). Gels were visualized in UVP Biospectrum AC Imaging System (UVP BioImaging Systems, Hanover, Germany) after they had been staining in 0.5 μg/mL EtBr (Sigma-Aldrich GmbH, Darmstadt, Germany) for 20 min. PCR amplifications were repeated twice for each primer combination to ensure the reproducibility of results.

### 4.6. FT-IR Spectroscopy Measurements

FT-IR assay was conducted using an FT-IR-4100 spectrophotometer (Jasco, Germany) according to the KBr pellet technique. The DNA samples were mixed with the KBr powder in a proportion of 1:100 and pressed subsequently to form a transparent pellet ready to use for the IR analysis. Each spectrum was recorded using a spectral resolution of 4 cm^−1^. All spectra were acquired over 256 scans. Profiles are presented in the wavenumber range 800–1800 cm^−1^.

### 4.7. Data Processing and Analysis

Morphological data analysis was carried out using SPSS version 19. First, one-way analysis of variance (ANOVA) was performed to compare the means of different morphological characters measured both in parents and hybrids. When the null hypothesis was rejected, Tukey’s post hoc test was applied at the *p* ≤ 0.05 significance level to determine statistically significant differences between the means. Values shown are means ± SD. The same lowercase letters indicate no significant differences between the means.

To present the morphological relationships between parents and F1 progenies, the Euclidean distance coefficient was used as implemented in the NTSYS-pc package 2.1 (SIMINT module). Genetic distance matrix was calculated by Euclidean distance and then used for UPGMA cluster analysis. Mantel’s (1967) test was computed to examine how well the cluster analysis fits the distance matrix using the COPH and MXCOMP modules.

SRAP gel images were analyzed using TotalLab TL120 software (Nonlinear Dynamics, Newcastle upon Tyne, UK) determining band sizes. Only distinct and reproducible bands of the electrophoretic profiles by selected SRAP marker combinations were scored as 1 (present) or 0 (absent) and transferred to a binary matrix using MS Excel. Polymorphism information content (PIC) was calculated using the formula published by Zheng et al. (2017) [27]: PIC = 1 − (*f*^2^ + (1 − *f*)^2^), where *f* is the frequency of the marker in the data set. Due to the fact that every SRAP primer combination generated multiple dominant bands, each band was considered as one locus for PIC calculation [60]. SRAP genetic distances were calculated using the Nei’s (1972) [30] similarity coefficient. An UPGMA [61] (pp. 39–42) dendrogram was also constructed based on a symmetric dissimilarity matrix using the SHAN module in NTSYS program, version 2.1 [62]. The COPH and MXCOMP modules of NTSYS 2.1 were used to check the goodness-of-fit between the cluster analysis and dissimilarity matrix. The generated FT-IR spectra were processed with the OriginPro 8.5.1 software (OriginLab, USA). Subsequent to the baseline correction, the FT-IR spectra were scaled to have equal intensity in the band at ≈ 1101 cm^−1^, which was assigned to the symmetric stretching vibrations of PO_2_^−^. The preprocessed spectral data were collected into an ASCII database (corresponding to 15 samples × 1038 variables) and used later for multivariate analysis of FT-IR spectroscopic data. PCA analysis was performed by using The Unscrambler X^®^ 10.4 software (CAMO Software, Oslo, Norway). Hierarchical clustering analysis (HCA) of Fourier-transform infrared spectra was performed using Matlab software (The MathWorks, Inc., USA). The dendrogram was generated using the intra-spectral Euclidean distance based on the Ward’s algorithm for clustering [63]. The correlation coefficient was defined as the linear correlation between the cophenetic distances obtained from the tree and the original distances used to construct the tree. A comparison between SRAP and FT-IR marker systems was made using Mantel test in GenAlEx 6.502 [64] with 9999 permutations.

## 5. Conclusions

In this study, morphological traits and two different DNA methods were used to assess genetic relationships between parents and F1 hybrids. The results show that both DNA-based methods can be useful for further research in future breeding programs of Streptocarpus. The results obtained with the Mantel test confirm that SRAP markers proved to be efficient for the authentication of F1 hybrids and parental discrimination based on profile of PCR-amplified bands and the valuable types of identified markers. Our results suggest that the amplification of open reading frames (ORF) by SRAP markers have the potential to strengthen the relationship between DNA polymorphisms and morphological traits that characterize Streptocarpus genitors and F1 hybrids. The FT-IR spectral analysis identified that the biochemical variability of the analyzed samples was based mainly on subtle changes in the base functional groups, base-sugar entities, and backbone structures of genomic DNA. Our results also indicate that FT-IR spectroscopy can be used for preliminary evaluation and prediction of DNA heterogeneity at the whole genome level.

To the best of our knowledge, this is the first report made on the discrimination of F1 hybrids at the level of genomic DNA from Streptocarpus plants using FT-IR techniques.

## Figures and Tables

**Table 1:** Colors and Royal Horticultural Society Color Chart (RHSCC) code of genitors and F1 progenies selected for sequence-related amplified polymorphism (SRAP) and Fourier-transform infrared (FT-IR) analyses.

Sample no.	Parents and F1 Progenies	Color	RHSCC Code
1	P1 (“Black Panther”)	dark violet, blue violet	83B, N88A
2	P2 (“Slumber Song”)	purple pink, yellow	N73A, 4A
3	P3 (“Snow White”)	white	155B
4	H1	violet	N82B
5	H2	pink violet	N77B
6	H3	pink purple, blue violet	72A, N88A
7	H4	violet	N82D
8	H5	dark violet, violet	83B, 82B
9	H6	purple pink, yellow, white	N74C, 6A, 155B
10	H7	blue violet	N88C
11	H8	pink violet, white	N77B, 155A
12	H9	violet	N89A
13	H10	blue violet, dark violet	N88C, 83B
14	H11	blue violet, violet	N88C, 82B
15	H12	blue violet, dark pink violet	N88A, N77A

**Table 2:** List of SRAP primer pairs and the number of scored bands used in Streptocarpus.

Primer Combination	Number of Total Bands	Size Range of Bands (bp)	Number of Polymorphic Bands	% of Polymorphism	PIC
**Me8-Em6**	11	487–1553	10	90.90	0.42
**Me8-Em2**	9	686–1949	8	88.88	0.34
**Me4-Em8**	13	109–1502	12	92.30	0.45
**Me1-Em8**	7	104–523	7	100	0.35
**Me6-Em1**	15	237–1697	14	93.33	0.39
**Me6-Em8**	11	211–1348	11	100	0.37
**Me1-Em2**	16	175–1712	15	93.75	0.48
**Me4-Em2**	14	219–1013	13	92.85	0.46
**Total**	96		90	-	-
**Mean**	12 ± 0.02		11.25 ± 0.06	93.75 ± 0.26	0.40 ± 0.07

**Table 3:** SRAP marker types and their genetic polymorphism in Streptocarpus hybrids from the two parental crosses (P1 × P2 and P1 × P3).

Type of SRAP Marker	Band Presence/ Absence in ♀ Parent	Band Presence/ Absence in ♂ Parent	Band Presence/ Absence in F1 Hybrid	Total Number of Occurrences for Each Type of SRAP Marker	
				P1 × P2	P1 × P3
**I**	+	+	+	96	59
**II**	+	−	+	16	58
**III**	−	+	+	31	19
**Total**				143	136

**Table 4:** Peak positions (cm^−1^) and tentative assignments of FT-IR absorbance bands for DNA from the Streptocarpus P1 × P2 parental cross and their corresponding hybrids.

Streptocarpus Parents and Hybrids								Tentative Assignment ^1^	References
*P1*	*P2*	*H1*	*H2*	*H3*	*H4*	*H5*	*H6*		
873	871	858	861	-	869	-	-	bk, deoxyribose, possible A-form	[31,32,33,34]
964	962	958	960	958	962	958	958	Furanose-phosphate skeletal motions	[35]
-	1024	1016	1024	1027	1018	1025	1020	Deoxyribose	[36]
-	1079	1082	1078	1081	1081	1078	1078	υ_s_PO_2_^−^	[31,37,38]
1103	1101	1103	1103	1101	1101	1101	1101	υ_s_PO_2_^−^	[37,38]
1151	1149	1147	1145	1151	1149	1151	1147	Deoxyribose, C3‘-*endo*/*anti*, A-form	[39]
1240	1242	1244	1244	1244	1243	1245	1245	υ_a_PO_2_^−^, possible A-form	[31,32,40]
1363	1377	1371	1372	1371	1363	1371	1363	dA, dG (C2′-*endo*/*anti*)	[32,39,41]
1415	1417	1419	1415	1417	1419	1417	1417	C2′-*endo*/*anti* sugar pucker (B-form)	[32,39]
1491	1489	-	-	1495	1487	1489	1487	Base ring modes	[42]
1609	1603	-	1614	-	1605	1599	1608	dA, possible C=C, C=N	[31,43,44]
-	1659	1653	1670	1649	-	1664	1670	dT (C=O)	[32]

^1^ Abbreviations: bk-backbone, υ-stretching vibration, dA-deoxyadenosine; dG-deoxyguanosine; dT-thymidine.

**Table 5:** Peak positions (cm^−1^) and tentative assignments of FT-IR absorbance bands for DNA from the Streptocarpus P1 × P3 parental cross and their corresponding hybrids.

Streptocarpus Parents and Hybrids								Tentative Assignment ^1^	References
*P1*	*P3*	*H7*	*H8*	*H9*	*H10*	*H11*	*H12*		
873	875	863	861	873	863	-	875	bk, deoxyribose, possible A-form	[31,32,33,34]
964	960	967	958	964	964	958	962	Furanose-phosphate skeletal motions	[35]
-	1023	1023	1020	1023	1024	1016	1018	Deoxyribose	[36]
1049	1076	1078	1078	1078	1078	1078	1081	υ_s_PO_2_^−^	[31,37,38]
1103	1103	1101	1101	1101	1101	1101	1101	υ_s_PO_2_^−^	[37,38]
1151	1145	1147	1145	1145	1147	1147	1145	Deoxyribose, C3‘-*endo*/*anti*, A-form	[39]
1240	1246	1243	1249	1244	1242	1240	1246	υ_a_PO_2_^−^, possible A-form	[31,32,40]
1363	1373	-	1373	1373	1367	1366	1376	dA, dG (C2′-*endo*/*anti*)	[32,39,41]
1415	1419	1417	1419	1417	1417	1417	1419	C2′-*endo*/*anti* sugar pucker (B-form)	[32,39]
1491	-	-	1491	1492	1492	1492	1492	Base ring modes	[42]
1609	1622	-	-	-	-	1607	-	dA, possible C=C, C=N	[31,43,44]
-	-	1657	-	1655	1647	-	1651	dT (C=O)	[32]

^1^ Abbreviations: bk-backbone, υ-stretching vibration, dA-deoxyadenosine; dG-deoxyguanosine; dT-thymidine.

**Table 6:** Forward and reverse SRAP primer sequences.

Primer	Sequences 3′-5′	Primer	Sequences 5′-3′
Me*1	TGAGTCCAAACCGGATA	**Em3**	GACTGCGTACGAATTGAC
**Me4**	TGAGTCCAAACCGGACC	**Em4**	GACTGCGTACGAATTTGA
**Me6**	TGAGTCCAAACCGGTAA	**Em5**	GACTGCGTACGAATTAAC
**Me8**	TGAGTCCAAACCGGTGC	**Em6**	GACTGCGTACGAATTGCA
Em**1	GACTGCGTACGAATTAAT	**Em7**	GACTGCGTACGAATTCAA
**Em2**	GACTGCGTACGAATTTGC	**Em8**	GACTGCGTACGAATTCTG

Me* and Em** represent forward and reverse primers.

## Supplementary Materials

The following are available online at https://www.mdpi.com/2223-7747/9/2/160/s1, Table S1: Morphological characterization of genitors and F1 progenies selected for SRAP and FT-IR analyses.
