# Supplementary material for: Assessment of Genetic Relationships between Streptocarpus x hybridus V. Parents and F1 Progenies Using SRAP Markers and FT-IR Spectroscopy
Source: Plants (Basel). 2020 Jan 28;9(2):160. doi: 10.3390/plants9020160 (PMC7076643; doi:10.3390/plants9020160)
Supplement: Supplementary file 1 [file plants-09-00160-s001.pdf]

Table S1. Morphological characterization of genitors and F1 progenies selected for SRAP and FT-IR. analyses

	Parents and F1 progenies	Number of flowers/ plant NF	Number of peduncle/plant NP	Number of flower/ penduncle NFP	Length of peduncle LP(cm)	Length of corolla tube LCT(cm)	Width of flower WF(cm)
1	P1 ('Black Panther')	20.47±0.37 [c]	8.27±0.20 [bcd]	2.48±0.08 [h]	18.92±0.01 [d]	3.79±0.03 [f]	6.26±0.03 [d]
2	P2 ('Slumber Song')	16.33±0.47 [a]	7.33±0.22 [a]	2.24±0.09 [bcd]	13.64±0.01 [b]	2.72±0.01 [b]	4.01±0.06 [b]
3	P3 ('Snow White')	18.07±0.43 [b]	10.27±0.20 [h]	1.77±0.07 [a]	8.82±0.03 [a]	2.08±0.02 [a]	3.05±0.02 [a]
4	H1	20.73±0.43 [c]	9.40±0.23 [g]	2.21±0.06 [bc]	17.54±0.20 [cd]	3.76±0.03 [ef]	6.61±0.08 [d]
5	H2	18.47±0.29 [b]	7.87±0.16 [abc]	2.35±0.05 [defg]	16.17±0.12 [c]	3.25±0.01 [c]	5.18±0.04 [c]
6	H3	20.87±0.33 [c]	8.60±0.23 [def]	2.43±0.07 [fgh]	18.99±0.03 [d]	3.84±0.02 [f]	6.34±0.03 [d]
7	H4	17.87±0.33 [b]	7.73±0.20 [ab]	2.32±0.07 [cdef]	16.21±0.17 [c]	3.35±0.02 [cd]	5.25±0.15 [c]
8	H5	20.07±0.40 [c]	9.33±0.28 [g]	2.16±0.09 [b]	18.13±0.11 [d]	3.73±0.04 [ef]	6.14±0.08 [d]
9	H6	16.40±0.23 [a]	7.40±0.23 [a]	2.23±0.07 [bc]	14.18±0.14 [b]	2.80±0.04 [b]	4.16±0.04 [b]
10	H7	20.60±0.33 [c]	8.40±0.23 [cde]	2.46±0.09 [gh]	18.61±0.02 [d]	3.68±0.04 [def]	6.07±0.02 [d]
11	H8	20.67±0.53 [c]	9.20±0.25 [g]	2.26±0.09 [bcd]	18.26±0.14 [d]	3.44±0.07 [cde]	6.61±0.07 [d]
12	H9	20.47±0.23 [c]	8.87±0.16 [efg]	2.31±0.06 [cde]	18.85±0.19 [d]	3.80±0.04 [f]	6.28±0.05 [d]
13	H10	21.07±0.31 [c]	9.13±0.29 [fg]	2.32±0.08 [cdef]	18.97±0.05 [d]	3.81±0.04 [f]	6.47±0.02 [d]
14	H11	20.53±0.37 [c]	8.53±0.23 [de]	2.41±0.07 [efgh]	18.73±0.19 [d]	3.71±0.02 [ef]	6.31±0.08 [d]
15	H12	20.67±0.32 [c]	9.33±0.22 [g]	2.22±0.06 [bc]	18.79±0.14 [d]	3.84±0.05 [f]	6.64±0.05 [d]

[1]The analyzed ornamental flower traits were: NF- number of flowers/plant, NP-number of peduncles/plant, NFP- number of flowers/peduncle, LP- length of peduncle (cm), LCT- length of corolla tube (cm) and WF- width of flower (cm). Values shown are means ±SD ($p \leq 0.05$). Lowercase letters in common indicate no significant differences between the means within the same character according to Tukey's post-hoc test ($P \leq 0.05$).

Supplementary Materials: The following are available online at www.mdpi.com/xxx/s1, Table S1: Morphological characterization of genitors and F1 progenies selected for SRAP and FT-IR analyses.
